# Unclaimed Prize Information Biases Perceptions of Winning in Scratch Card Gambling

**DOI:** 10.1007/s10899-018-9770-2

**Published:** 2018-03-29

**Authors:** Alexander C. Walker, Madison Stange, Jonathan A. Fugelsang, Derek J. Koehler, Mike J. Dixon

**Affiliations:** Department of Psychology, University of Waterloo, Waterloo, ON N2L 3G1 Canada

**Keywords:** Scratch cards, Cognitive biases, Decision making, Unclaimed prize information, Gambling

## Abstract

Unclaimed prize information (i.e., the number of prizes still available to be won) is information commonly provided to scratch card gamblers. However, unless the number of tickets remaining to be purchased is also provided, this information is uninformative. Despite its lack of utility in assisting gamblers in choosing the most favourable type of scratch card to play, we hypothesized that unclaimed prize information would bias participants’ judgments within a scratch card gambling context. In Experiment 1 (*N* = 201), we showed that participants are influenced by this information such that they felt more likely to win, were more excited to play, and preferred to hypothetically purchase more of the scratch card with the greatest number of unclaimed prizes. In Experiment 2 (*N* = 201), we attempted to ameliorate this bias by providing participants with the number of tickets remaining to be purchased and equating the payback percentages of all three games. The bias, although attenuated, still persisted in these conditions. Finally, in Experiment 3 (*N* = 200), we manipulated the hypothetical scratch cards such that games with the highest number of unclaimed prizes were the least favourable, and vice versa. As in Experiment 2, participants still favoured cards with greater numbers of unclaimed prizes. Possible mechanisms underlying this bias are discussed. In conclusion, across three experiments, we demonstrate that salient unclaimed prize information is capable of exerting a strong effect over judgments related to scratch card games.

## Introduction

Part of making informed decisions involves disregarding uninformative information. Research on human decision-making has indicated that individuals can be unduly influenced by irrelevant information (Ariely et al. 2003; Tversky and Kahneman 1974; Van Osselaer et al. 2004). For example, a seminal paper by Tversky and Kahneman (1974) found that participants’ judgments of the percentage of African countries in the United Nations was shown to be unduly biased by an arbitrary number obtained from spinning a “wheel of fortune.” Similarly, in a study conducted by Ariely et al. (2003), the magnitude of participant’s last two-digits of their social security number was found to impact their willingness-to-pay (WTP) for various consumer products. The above findings exemplify the use of a heuristic in which judgments are made by anchoring to a value and then adjusting away from this value (often insufficiently).

Importantly, the anchoring heuristic is not the only way individuals incorporate uninformative information into their decisions. Research in the field of consumer psychology has demonstrated that irrelevant product attributes influence consumer choice (Carpenter et al. 1994; Meyvis and Janiszewski 2002; Van Osselaer et al. 2004). Additionally, simply repeating statements has been shown to make them appear more believable to participants compared to their non-repeated counterparts (Fazio et al. 2015; Hasher et al. 1977). Furthermore, merely altering the way a problem is framed can impact decision making (Tversky and Kahneman 1981). Lastly, people have been shown to be biased towards information gain, such that they will endure costs to receive even inconsequential information (Baron et al. 1988).

People may be especially likely to incorporate irrelevant information into their decision making when this information is salient and holds intuitive appeal. Research on ratio bias has demonstrated that people tend to favour intuitive alternatives, even when these alternatives are suboptimal (Denes-Raj and Epstein 1994; Denes-Raj et al. 1995; Kirkpatrick and Epstein 1992; Miller et al. 1989). In the traditional ratio bias paradigm participants attempt to select a “winning” red jelly bean from a bowl featuring both red and white jelly beans. Importantly, participants are given two bowls to choose from: a large bowl that contains several red jelly beans (e.g., 7) with a small ratio of red to white beans (e.g., 7:93), and a small bowl that contains a single red jelly bean with a larger ratio of red to white beans (e.g., 1:9) and therefore a greater chance of winning compared to the large bowl. Despite the small bowl affording the best chance of selecting a red jelly bean, participants frequently will select from the large bowl (Denes-Raj and Epstein 1994). Thus, the strong intuitive appeal of selecting from the bowl with the greatest number of winning jelly beans leads people to make a statistically suboptimal choice. Participant’s self-reports showed that this bias occurred even for participants who were aware that the small bowl was the optimal choice.

In a medical context, Garcia-Retamero et al. (2010) observed a ratio bias when investigating people’s ability to interpret risks accurately. Here, participants were overly influenced by how often an event happened (e.g., the number of treated and non-treated patients who die) while being insufficiently influenced by the total number of opportunities an event had to occur (e.g., the overall number of treated and non-treated patients). Overall, highly intuitive, uninformative information can bias individuals, even in the presence of informative information.

A real-world context in which cognitive biases can be observed is in lottery gambling scenarios. For example, regular lottery gamblers are influenced by how “random” lottery numbers appear to be (i.e., their “representativeness”; Rogers and Webley 2001). Similarly, participants will choose lottery numbers that appear to be more random (e.g., have fewer repeating digits), lending additional support for the representativeness heuristic in lottery gambling decisions (Holtgraves and Skeel 1992). Past research has also demonstrated that lottery gamblers will generally prefer the number seven, small numbers, and will avoid large numbers and adjacent numbers (Turner 2010). Collectively, these findings suggest that lottery gamblers’ cognitions can be unduly biased by information that they encounter and beliefs that they hold, despite the fact that winning numbers chosen for traditional lottery draws are random and no strategy can aid in choosing a winning combination. The current study seeks to explore whether such cognitive biases also occur among those who play scratch cards, a relatively unexplored area of gambling research.

### Unclaimed Prize Information

Some sources of information provided with scratch card games have no bearing on the outcomes of the games themselves. One such source is unclaimed prize information, which informs gamblers of the number of prizes available to be won. This information is available to players online through all Canadian lottery operators with most operators updating this information weekly or daily (Atlantic Lottery Corporation 2018; British Columbia Lottery Corporation 2018; Lotto-Quebec 2018; Ontario Lottery and Gaming Corporation 2018; Western Canada Lottery Corporation 2018). For each scratch card game in circulation, a table is presented to players that includes a count of the total number of each prize (present at the outset of the game) and the number of these prizes that remain unclaimed. At first blush, unclaimed prize information *feels* intuitively useful—certainly purchasing cards with more prizes remaining allows for the greatest likelihood of winning. However, if the number of prizes remaining is the *only* information provided, this information is uninformative to the lottery player hoping to maximize their probability of success.

In order to maximize their chances of monetary gain, scratch card gamblers should choose to play games with the highest payback percentages, defined as the amount of money bet on a certain game that is paid out as prizing (Harrigan et al. 2011). This value is calculated by dividing the total value of all prizes by the cost of all scratch cards remaining. Therefore, unclaimed prize information, when presented *without* the number of cards remaining in circulation, is not useful in the sense that it cannot be used to calculate the current payback percentage of a scratch card game. Consequently, gamblers cannot use unclaimed prize information to ensure that they are consistently playing the most valuable games. For example, unclaimed prize information may indicate that Scratch Card A has four $1000 prizes remaining while Scratch Card B possesses a single $1000 prize. This information alone does not allow one to conclude that Scratch Card A is of a higher value due to the fact that Scratch Card A may have far more cards in circulation compared to Scratch Card B, and thus offer a far lower chance at a $1000 prize. Using this example, it is evident that when unclaimed prize information is used independently, it is valueless to the gambler looking to increase their chances for monetary gain. This of course assumes that all gamblers’ primary motivation is to maximize their likelihood of monetary gain. It is possible that some gamblers may be motivated by the opportunity of winning a particular prize. In this case, unclaimed prize information may help gamblers avoid games that no longer offer the possibility of a specific desired prize. Nevertheless, it appears that gamblers attempt to use this information to maximize monetary gain, as exemplified in Ontario-based online resources that attempt to make scratch card recommendations on the basis of unclaimed prize information (Opid Technologies 2017; Usockem 2016).

### Current Study

It is unknown whether variation in unclaimed prize information influences gambler’s judgments in scratch card gambling scenarios. Research on cognitive biases suggest that people can be influenced by uninformative information when making decisions (Ariely et al. 2003; Tversky and Kahneman 1974; Van Osselaer et al. 2004). Therefore, it is possible that people may be unduly influenced by unclaimed prize information. Support for this claim comes from research on ratio-bias, in which the numerator of a ratio has shown to be overvalued (Denes-Raj and Epstein 1994; Denes-Raj et al. 1995; Garcia-Retamero et al. 2010; Kirkpatrick and Epstein 1992). In terms of scratch card gambling, it is possible that a similar phenomenon occurs with the presence of unclaimed prize information, such that this salient information is overvalued despite being uninformative in its current presentation.

The current study seeks to examine the potential biasing effects of unclaimed prize information in scratch card gambling scenarios. To this end, we are interested in how varying amounts of unclaimed prizes will impact how likely players believe they are to win on a scratch card game, how excited they think they would be when playing the games, and how many of each card participants would elect to purchase in a hypothetical gambling scenario. Across three experiments, we predict that participants will be influenced by unclaimed prize information such that their judgments and decisions will be yoked to the number of unclaimed prizes presented.

## Experiment 1

The primary goal of Experiment 1 was to investigate the influence of unclaimed prize information on participants’ judgments regarding scratch card gambling. Specifically, we hypothesized that participants would feel that they were more likely to win, be more excited to play, and ultimately choose to hypothetically purchase scratch cards featuring higher numbers of unclaimed prizes.

## Method

### Participants

A sample of 201 participants was recruited from Amazon Mechanical Turk to complete an online questionnaire. All descriptive statistics for this sample can be viewed in Table 1. Participants were recruited under the condition that they be U.S. residents and possess a Mechanical Turk HIT approval rate greater than or equal to 95%. The present experiment took approximately 20 min to complete and participants were compensated $3.00 for their participation. All reported experiments received prior approval by a University of Waterloo Research Ethics Committee.

**Table 1:** Descriptive statistics

Measure	Experiment 1	Experiment 2	Experiment 3
Age, mean (SD)	33.20 (9.0)	34.42 (11.33)	32.92 (8.60)
Gender, % females	37.3%	44.8%	33.8%
Frequency of scratch card gambling, *n* (%)			
Had not played	66 (33.2%)	65 (32.5%)	59 (29.5%)
1–5 times	74 (37.2%)	66 (33.0%)	74 (37.0%)
6–10 times	28 (14.1%)	26 (13.0%)	22 (11.0%)
11–15 times	12 (6.0%)	15 (7.5%)	19 (9.5%)
16–24 times	8 (4.0%)	7 (3.5%)	10 (5.0%)
24 or more	11 (5.5%)	21 (10.5%)	16 (8.0%)
Problem Gambling Severity Index, *n* (%)			
Non-problem gambling	125 (62.8%)	111 (57.2%)	96 (49.2%)
Low-risk gambling	54 (27.1%)	47 (24.2%)	59 (30.3%)
Moderate-risk gambling	7 (3.5%)	11 (5.7%)	13 (6.7%)
Problem gambling	13 (6.5%)	25 (12.9%)	27 (13.8%)

Descriptive statistics for all measures presented in Experiments 1–3. Categories for the Frequency of Scratch Card Gambling represent participants’ self-reported scratch card gambling frequency in the last 12 months

Problem Gambling Severity Index category cut-offs are based on those provided by Currie et al. (2013)

### Materials

#### Scratch Card Games

An image of a representative scratch card (100X Multiplier) was chosen from the Ontario Lottery and Gaming Corporation’s (OLG) website. Using Adobe Photoshop CS6, three versions of the same card were created (Green, Blue, and Red) by changing the colour of the scratch card. Specific information related to the number of top prizes and odds was also removed from the card image as to not conflict with information that was presented within the experiment.

#### Likelihood of Winning

At various time points, participants rated their likelihood of winning any prize while playing 100X Multiplier by responding to the following item: “How likely do you think you are to win a prize while playing 100X Multiplier?” Participants responded to this item using a scale that ranged from 1 (*Extremely unlikely*) to 7 (*Extremely likely*). Participants were polled following an introduction to the scratch card game (baseline) and then again following the presentation of unclaimed prize information for each game version.

#### Perceived Excitement

Participants responded to the following item: “How excited would you be to play 100X Multiplier?” Participants rated each version of the game on a scale from 1 (*Not at all excited*) to 7 (*Extremely excited*). These ratings were made following the presentation of specific card information containing details about the number of unclaimed prizes.

#### Card Preference

In order to assess participants’ card preferences, a hypothetical scenario was constructed in which participants were asked to imagine that they possessed a $25.00 gift card which could be used to purchase the presented scratch card games. Each available scratch card cost $5.00 to purchase and thus, participants were asked to purchase a total of five scratch card games. Participants indicated how many of each version of 100X Multiplier (Green, Blue, Red, each featuring either a low, medium, or high number of unclaimed prizes) they would elect to purchase in this hypothetical scenario. This item was a forced-choice question, in which participants had to purchase a total of five scratch cards before proceeding in the experiment.

#### Willingness-to-Pay (WTP)

Participants were asked the following: “Assuming you were going to purchase 5 of these scratch cards, how much money would you be willing to pay for unclaimed prize information for all 3 versions of 100X Multiplier?” Participants were asked to provide a dollar amount in an open text-box, which allowed a free text response.

### Design

Participants were randomly assigned to one of three conditions. Each condition differed in terms of which version of 100X Multiplier (Green, Blue, or Red) contained which amount of unclaimed prizes (low, medium, or high). This counterbalancing ensured that any colour preferences that may be present in the sample were controlled for. Additionally, the order in which the unclaimed prizes were presented was counterbalanced, such that each amount of unclaimed prizes (low, medium, or high) was presented first, second, and third.

### Procedure

To begin the experiment, participants were given a description of the scratch cards that would be used. Three simplified scratch card game images, based on a scratch card game available for sale in our home jurisdiction of Ontario (“100X Multiplier”; OLG 2017), were presented as hypothetical games to participants. Information regarding each game’s cost, total number of prizes, and top prize amount was presented along with instructions on how to play each hypothetical game. Each scratch card game was identical with regards to this presented information. However, crucially, participants were told that even though the three versions of the game (Green, Blue, and Red) had the same cost and prize distribution, they could differ with regards to the number of cards remaining to be purchased, and the number of prizes remaining to be won. Following this information, participants were asked to make a baseline judgment of how likely they felt they were to win a prize while playing any of the 100X Multiplier games.

Following this introductory phase, participants were given prize amounts, total prize, and unclaimed prize information for each 100X Multiplier game (Green, Blue, Red). Participants were informed that “total prize” information referred to the total number of each prize that exists out of all of the scratch cards that were printed while “unclaimed prize” information referred to the number of each prize that was still available to be won. To convey this information, an image of each game was presented with a table featuring the following headings: Game, Prize Amount, Total Prizes, and Unclaimed Prizes (see Table 2). Each game featured the same prize amounts (i.e., $1,000,000, $25,000 and $1000) as well as the same number of total prizes (two $1,000,000 prizes, ten $25,000 prizes, and 100 $1000 prizes). The critical feature on which the games differed was the number of unclaimed prizes for each prize type. The numbers of unclaimed prizes for each game were chosen so that one game had approximately 75% of each prize remaining (high amount of unclaimed prizes), one game had approximately 50% of each prize remaining (medium amount of unclaimed prizes), and the final game had approximately 25% of each prize remaining (low amount of unclaimed prizes). The exact unclaimed prize values for games featuring high, medium, and low unclaimed prize amounts can be seen in Table 2. After being presented with the information for one 100X Multiplier game, participants made two judgments: one regarding their likelihood of winning a prize while playing the game in question, and the other about how excited they would be to play the game. Overall, participants made six judgments regarding their likelihood of winning and perceived excitement (two judgments per game).

**Table 2:** An example of scratch card information provided to participants in Experiment 1

Card type	Game	Prize amount	Total prizes	Unclaimed prizes
High unclaimed prizes	100X Multiplier (green)	$1,000,000	2	2
	100X Multiplier (green)	$25,000	10	7
	100X Multiplier (green)	$1000	100	78
Medium unclaimed prizes	100X Multiplier (blue)	$1,000,000	2	1
	100X Multiplier (blue)	$25,000	10	5
	100X Multiplier (blue)	$1000	100	54
Low unclaimed prizes	100X Multiplier (red)	$1,000,000	2	0
	100X Multiplier (red)	$25,000	10	3
	100X Multiplier (red)	$1000	100	24

Following these judgments, participants were again presented with a table containing prize amount, total prize, and unclaimed prize information for each scratch card game. After being presented with this information, participants were asked to make a WTP judgment. Specifically, participants were asked to assume that they were purchasing five of the 100X Multiplier scratch cards, and to report how much money they would be willing to pay for the unclaimed prize information of all three versions of 100X Multiplier. Next, participants were given a hypothetical scenario (see card preference) and asked how many of each 100X Multiplier game they would elect to purchase (summing to a total of five games). For reference, participants were again given all unclaimed prize information when making their decision.

Finally, participants completed a number of scales and questionnaires. First, participants completed a set of demographic questions pertaining to their age, gender, and scratch card gambling frequency. Next, participants were asked if they felt like they were missing any pieces of information that would have been helpful in making their decisions about the scratch cards presented in the study. Lastly, participants completed a set of three modified Cognitive Reflection Test (CRT; Frederick 2005) items, nine Problem Gambling Severity Index (PGSI; Ferris and Wynne 2001) items, as well as a standard base-rate neglect task. The CRT items and base-rate neglect task were administered for reasons peripheral to the current study.

## Results

### Likelihood of Winning

**Fig. 1:**
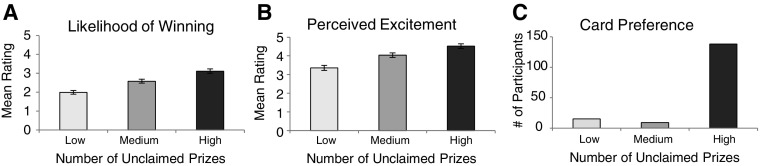
Results from Experiment 1. **a** Mean likelihood of winning values for low, medium, and high unclaimed prize levels. **b** Mean perceived excitement values for low, medium, and high unclaimed prize levels. **c** Mean number of cards chosen for low, medium, and high unclaimed prize levels. All error bars are ± 1 SE

Participants’ ratings of how likely a win was on the various 100X Multiplier cards were averaged and compared with a repeated-measures analysis of variance (ANOVA). In cases where the sphericity assumption was violated, a Greenhouse–Geisser correction was applied to the degrees of freedom. The overall ANOVA revealed a main effect of unclaimed prize information, *F*(1.69, 335.04) = 69.106, *p* < .001, $\eta _{p}^{2}$ = .259 (see Fig. 1a). Follow-up paired samples *t*-tests revealed significant differences between participant’s likelihood ratings for games with a high number of unclaimed prizes (*M* = 3.12, SD = 1.59) and a medium number of unclaimed prizes (*M* = 2.58, SD = 1.49), *t*(198) = 6.30, *p* < .001, significant differences between a medium number of unclaimed prizes and a low number of unclaimed prizes (*M* = 2.00, SD = 1.35), *t*(198) = 6.95 *p* < .001, and significant differences between a high number of unclaimed prizes and a low number of unclaimed prizes, *t*(200) = 9.82, *p* < .001. Additionally, further paired samples *t*-tests comparing participants’ likelihood ratings for scratch cards featuring high, medium, and low amounts of unclaimed prizes to participants’ baseline likelihood ratings (i.e., ratings made prior to the presentation of unclaimed prize information) revealed significant differences. Specifically, participants’ likelihood ratings for cards featuring high amounts of unclaimed prizes was shown to be significantly higher than baseline likelihood ratings (*M* = 2.58, SD = 1.46), *t*(200) = 5.00, *p* < .001. In contrast, likelihood ratings given to cards featuring low amounts of unclaimed prizes were revealed to be significantly lower than baseline likelihood ratings, *t*(200) = − 6.32, *p *< .001. Finally, likelihood ratings given to cards with a medium amount of unclaimed prizes did not differ from baseline likelihood judgments (*p *> .05).

### Perceived Excitement

Participants’ ratings of perceived excitement for playing the given scratch card games were averaged and compared with a repeated measures ANOVA. The overall ANOVA revealed a main effect of unclaimed prize information, *F*(1.69, 337.05) = 52.80, *p* < .001, $\eta _{p}^{2}$ = .210 (see Fig. 1b). Follow-up paired samples *t*-tests revealed significant differences between excitement ratings when there was a high number of unclaimed prizes (*M* = 4.52, SD = 1.70) and a medium number of unclaimed prizes (*M* = 4.04, SD = 1.72), *t*(199) = 4.96, *p* < .001, when there was a medium number of unclaimed prizes and a low number of unclaimed prizes (*M* = 3.36, SD = 1.91), *t*(199) = 6.50, *p* < .001, as well as when there was a high number of unclaimed prizes and a low number of unclaimed prizes, *t*(199) = 8.58, *p* < .001.

### Card Preference

In the hypothetical card purchasing data, we were constrained in the types of analyses we could conduct by the deterministic nature of the question design and resulting data (if a participant’s purchases were two “high” cards and two “medium” cards then they *must* have purchased one “low” card). Such determinism would violate assumptions of parametric and non-parametric statistical tests. Participants’ card preferences were therefore assessed by determining which card each participant preferred (i.e., the card that they hypothetically purchased the most). To operationalize card preference, we used the mode of hypothetical purchases. For example, if a participant bought three cards with a high number of unclaimed prizes and one each of medium and low unclaimed prizes, this participant was deemed to prefer the cards with a high number of unclaimed prizes (i.e., the mode for their purchases was associated with the “high” category). If a player distributed their purchases such that there were two modes (e.g., two “high”, two “medium”, and one “low” card) they were deemed not to have shown a clear preference for any single type of card. In our sample there were 38 such participants who showed no clear preference. Of the remaining 162 who showed a clear card preference (i.e., who had a single mode), 138 participants preferred cards with a high number of unclaimed prizes. Nine participants showed a preference for scratch cards with a medium number of unclaimed prizes, and 15 participants showed a preference for scratch cards with a low number of unclaimed prizes (see Fig. 1c). We compared the data of these participants (who showed unimodal preferences) using Chi square goodness-of-fit tests, in which the number of participants who established a card preference (calculated for each card type), were compared to expected values in which no preference was assumed across the cards in the comparison (i.e., equal preference between all card types involved in the analysis).

First, we conducted a test to determine if the observed preferences among all three card types differed from the preferences that would be expected if participants were not influenced by unclaimed prize information in our hypothetical purchasing task. This test revealed an influence of unclaimed prize information as observed preferences significantly differed from the preferences one would expect given no influence of unclaimed prizes, *X*^2^(2) = 196.33, *p* < .001. Next, we conducted three further Chi square goodness-of-fit tests comparing participants’ preferences for high versus medium, high versus low, and medium versus low unclaimed prize cards, compared to expected equal preferences between these pairs. These analyses revealed that participants preferred to hypothetically purchase cards with a high amount of unclaimed prizes compared to cards featuring a low [*X*^*2*^(1) = 49.44, *p* < .001] and medium [*X*^*2*^(1) = 56.60, *p* < .001] amount of unclaimed prizes. Furthermore, participants showed no preference between cards with medium and low numbers of unclaimed prizes, *X*^*2*^(1) = .75, *p* > .05.

### Willingness to Pay

Participant responses were first cleaned to ensure a consistent format (e.g., converting written amounts into numerical amounts, removing dollar signs, etc.). The median willingness to pay value reported was $5.00, and responses ranged from $0.00 to $5000. Importantly, only 21 participants (10.4%) indicated that they would be unwilling to pay for unclaimed prize information (i.e., input $0 as their response).

## Discussion

Despite its lack of utility, the results of Experiment 1 strongly suggest that unclaimed prize information influences participants’ judgments of scratch card games as well as their behaviours in a gambling context. Specifically, participants were found to judge scratch cards with greater numbers of unclaimed prizes as being more likely to lead to a win compared to cards with fewer unclaimed prizes. Similarly, participants were more excited to play and more likely to hypothetically purchase scratch cards featuring greater numbers of unclaimed prizes in comparison to those featuring fewer unclaimed prizes. In line with these findings, only 10.4% of participants indicated that they would be unwilling to pay for access to unclaimed prize information, with the majority of participants valuing this information at or above $5.00. Overall, these results suggest that unclaimed prize information, provided to players by various lottery operators, is capable of having significant influence over gamblers’ subjective feelings of winning, excitement, and scratch card purchasing behaviours.

### Experiment 2

Having demonstrated the influence of unclaimed prize information in Experiment 1, we sought to attenuate this influence in Experiment 2 by providing participants with the information necessary to calculate the value of each presented scratch card game. Therefore, we provided participants with the total number of tickets remaining for each game in conjunction with unclaimed prize information as presented in Experiment 1. The presented number of tickets remaining for each game was constructed such that all three games possessed equal payback percentages. Thus, participants making use of the provided information should not be unduly biased by unclaimed prize information.

## Method

### Participants

A sample of 201 participants was recruited from Amazon Mechanical Turk and received $4.00 upon completion of a 25-min online questionnaire. All participants were recruited under the condition that they be U.S. residents and have a 95% (or greater) HIT approval rate on Mechanical Turk. Twenty participants were non-naïve to the experimental design (i.e., they also participated in Experiment 1). However, our results were not affected by the presence of the non-naïve participants (i.e., the interpretation of all significance tests were the same with non-naïve participants removed), and therefore we elected to retain our full sample.

### Materials

All materials were identical to those described in Experiment 1 with the following exception.

#### Calculating Payback Percentage

Unlike participants in Experiment 1, participants in Experiment 2 were given the information necessary to calculate a payback percentage for each 100X Multiplier scratch card. Thus, unlike in Experiment 1, we asked participants to demonstrate their ability to properly calculate the payback percentage of a scratch card game. Prior to asking participants to perform this calculation, participants were presented with a paragraph that explained what payback percentage represents in the context of a scratch card game. Next, participants were asked if they would be able to properly calculate the payback percentage of a scratch card game, given that they had all the necessary information. Finally, participants were given all relevant information for a scratch card game and asked to calculate the correct payback percentage for this game. Those who attempted this calculation provided their answer in a free-entry text box.

### Procedure

Experiment 2 followed a similar procedure to that described in Experiment 1 with the notable addition of the “total number of tickets remaining”. We provided participants with this information in order to investigate whether participants would still be biased by unclaimed prize information when they possessed the necessary information to calculate each game’s payback percentage. This information was presented alongside each game’s unclaimed prize information and was present during participants’ likelihood, excitement, card preference, and WTP judgments. Importantly, the total number of tickets remaining was such that each scratch card game featured an identical payback percentage of 70% (e.g., on average, $0.70 returned for each $1.00 bet). Specifically, games featuring a high amount unclaimed prizes featured 643,714 tickets remaining, games featuring a medium amount of unclaimed prizes featured 336,857 tickets remaining, and games featuring a low amount of unclaimed prizes featured 28,284 tickets remaining (see Table 3). Following the experimental task, participants’ ability to correctly calculate payback percentage was assessed.

**Table 3:** An example of scratch card information provided to participants in experiment 2

Card type	Game	prize amount	Total prizes	Unclaimed prizes
High unclaimed prizes	100X Multiplier (green)	$1,000,000	2	2
	100X Multiplier (green)	$25,000	10	7
	100X Multiplier (green)	$1000	100	78
	Total number of tickets remaining: 643,714			
Medium unclaimed prizes	100X Multiplier (blue)	$1,000,000	2	1
	100X Multiplier (blue)	$25,000	10	5
	100X Multiplier (blue)	$1000	100	54
	Total number of tickets remaining: 336,857			
Low unclaimed prizes	100X Multiplier (red)	$1,000,000	2	0
	100X Multiplier (red)	$25,000	10	3
	100X Multiplier (red)	$1000	100	24
	Total number of tickets remaining: 28,284			

## Results

### Likelihood of Winning

**Fig. 2:**
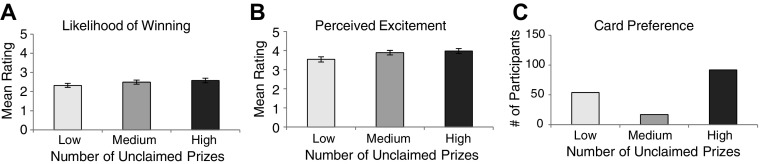
Results from Experiment 2. **a** Mean likelihood of winning values for low, medium, and high unclaimed prize levels. **b** Mean perceived excitement values for low, medium, and high unclaimed prize levels. **c** Mean number of cards chosen for low, medium, and high unclaimed prize levels. All error bars are ± 1 SE

Participants’ ratings of how likely a win was on the various 100X Multiplier cards were averaged and compared with a repeated-measures analysis of variance (ANOVA). The overall ANOVA revealed a main effect of unclaimed prize information, *F*(1.58, 316.07) = 3.653 *p* < .05, $\eta _{p}^{2}$ = .018 (see Fig. 2a). Follow-up paired samples * t*-tests revealed a significant difference between participants’ likelihood ratings for games featuring a high number of unclaimed prizes (*M* = 2.58, SD = 1.65) compared to a low number of unclaimed prizes (*M* = 2.32, SD = 1.51), *t*(200) = 2.19, *p* = .030. No significant differences were found when comparing participant’s likelihood ratings given for games featuring a medium number of unclaimed prizes (*M* = 2.50, SD = 1.54) to those given for games featuring either a high or low amount of unclaimed prizes (*p *> .05). Moreover, we once again used paired samples *t*-tests to compare participants’ likelihood ratings for scratch cards featuring high, medium, and low amounts of unclaimed prizes with participants’ baseline likelihood ratings (i.e., ratings made prior to the presentation of unclaimed prize information). As in Experiment 1, likelihood ratings given to scratch cards featuring low amounts of unclaimed prizes were significantly lower than participants’ baseline ratings (*M *= 2.63, SD = 1.58), *t*(200) = − 3.03, *p* = .003. All other comparisons with participants’ baseline likelihood ratings failed to reach significance (*p* > .05).

### Perceived Excitement

Participants’ ratings of perceived excitement for playing the given scratch card games were averaged and compared with a repeated measures ANOVA. The overall ANOVA revealed a main effect of unclaimed prize information, *F*(1.54, 304.25) = 9.74, *p* < .001, $\eta _{p}^{2}$ = .047 (see Fig. 2b). Follow-up paired samples *t*-tests revealed significant differences between excitement ratings when there was a high number of unclaimed prizes (*M* = 3.98, SD = 1.81) and a low number of unclaimed prizes (*M* = 3.54, SD = 1.90), *t*(198) = 3.39, *p* = .001, as well as a significant difference between excitement ratings when there was a medium number of unclaimed prizes (*M* = 3.89, SD = 1.69) and a low number of unclaimed prizes, *t*(198) = 3.59, *p* < .001. Finally, there was no significant difference between excitement ratings when participants were rating a card with a high number of unclaimed prizes versus when they were rating a card with a medium number of unclaimed prizes, *t*(200) = 1.09, *p* = .276.

### Card Preference

As in Experiment 1, participant’s card preferences were assessed by determining which card each participant preferred (i.e., the card that they hypothetically purchased the most). Thirty-eight participants did not establish a clear preference in favour of one card (i.e., split their preference between two cards equally) and therefore were not included in the subsequent analyses. Of the remaining 163 who showed a clear card preference, 92 preferred cards with a high number of unclaimed prizes. Seventeen participants showed a preference for scratch cards with a medium number of unclaimed prizes, and 54 participants showed a preference for scratch cards with a low number of unclaimed prizes (see Fig. 2c). To compare the number of cards purchased between each of the unclaimed prize categories, we conducted Chi square goodness-of-fit tests, examining the number of participants who established a card preference, (calculated for each card type) against a theoretical expected number of preferred cards assuming no participant preferences (i.e., equal preference between all card types involved in the analysis).

First, we conducted a test to determine if the observed preferences among all three card types differed from the preferences that would be expected if participants were not influenced by unclaimed prize information. Once again, this test revealed an influence of unclaimed prize information as observed preferences significantly differed from the preferences one would expect given no influence of unclaimed prizes, *X*^2^(2) = 51.77, *p* < .001. Additionally, we conducted three further Chi square goodness-of-fit tests comparing participants’ preferences for high versus medium, high versus low, and medium versus low unclaimed prize cards, compared to expected equal preferences between these pairs. These analyses revealed that participants preferred to hypothetically purchase cards with a high amount of unclaimed prizes compared to cards featuring a low [*X*^*2*^(1) = 4.95, *p* < .05] and medium [*X*^*2*^(1) = 25.80, *p* < .001] amount of unclaimed prizes. Furthermore, participants preferred cards with low numbers of unclaimed prizes compared to those with medium numbers of unclaimed prizes, *X*^*2*^(1) = 9.64, *p* < .01.

### Willingness to Pay

The median willingness to pay value reported was $5.00 with responses ranging from $0.00 to $10,000. Notably, only 17 participants (8.5%) indicated that they would be unwilling to pay for unclaimed prize information (i.e., input $0 as their response).

### Calculating Payback Percentage

When asked if, given the necessary information, they would be able to properly calculate the payback percentage of a scratch card game, 137 participants (68.5%) reported that they were able to do so. However, when asked to demonstrate this ability, only 30 participants (14.9%) correctly reported the payback percentage of the presented scratch card game.

## Discussion

Despite having all the information necessary to determine that the payback percentage of each game was identical, participants were still biased by unclaimed prize information. Replicating the results of Experiment 1, participants rated their likelihood of winning along with their perceived excitement as greater for scratch cards featuring larger amounts of unclaimed prizes. Furthermore, participants were once again most likely to hypothetically purchase the scratch card with the highest amount of unclaimed prizes. However, some previously significant differences between varying levels of unclaimed prizes failed to reach significance in Experiment 2 (e.g., likelihood of winning judgments for medium unclaimed prizes with both low and high unclaimed prize amounts; perceived excitement judgments for medium unclaimed prize amounts compared to high unclaimed prize amounts). Additionally, effect sizes for the aforementioned dependent variables notably decreased from Experiment 1 to Experiment 2 (reductions in effect sizes for all three experiments can be seen in Table 5). In sum, although it appears that providing participants with the total number of tickets remaining reduces the influence of unclaimed prize information, it does not *eliminate* this influence.

## Experiment 3

In Experiment 2 participants were provided with all the necessary information to calculate the payback percentage of each scratch card game. However, because all three scratch card games possessed identical payback percentages, participants may have still demonstrated a preference for games featuring higher amounts of unclaimed prizes when forced to choose between scratch cards in our hypothetical purchasing scenario. To eliminate this possibility, ticket remaining information was manipulated in Experiment 3 such that scratch cards with a low number of unclaimed prizes now featured the highest payback percentage; conversely, scratch cards with a high number of unclaimed prizes now featured the lowest payback percentage. Therefore, in Experiment 3, cards with low unclaimed prizes were the most valuable and thus should be preferred by participants.

## Method

### Participants

A sample of 200 participants was recruited from Amazon Mechanical Turk and received $4.00 upon completion of a 25-min online questionnaire. All participants were recruited under the condition that they be U.S. residents and have a 95% (or greater) HIT approval rate on Mechanical Turk. Of the 200 participants who completed Experiment 3, 43 participants had previously participated in Experiments 1 or 2. All analyses were conducted with both the non-naïve participants removed and retained. Only the likelihood of winning judgments were impacted by the inclusion of non-naïve participants. The updated results are presented in a footnote in the likelihood of winning results section.

### Materials

All materials were identical to those used in Experiment 2 with the exception that Experiment 3 featured the addition of the 7-item Actively Open-Minded Thinking Scale (AOT; Haran et al. 2013). As with the CRT and base-rate neglect task in Experiments 1 and 2, the AOT was administered for reasons peripheral to the current manuscript.

### Procedure

Experiment 3 followed an identical procedure to Experiments 1 and 2, with one notable exception. First, we changed the total number of tickets remaining for each scratch card game such that games possessing lower amounts of unclaimed prizes contained higher payback percentages compared to those possessing greater amounts of unclaimed prizes. Specifically, games that featured a low amount of unclaimed prizes now featured 26,400 tickets remaining and possessed a payback percentage of 75%, games featuring a medium amount of unclaimed prizes featured 336,857 tickets remaining and possessed a payback percentage of 70%, and games featuring a high amount of unclaimed prizes featured 693,231 tickets remaining and possessed a payback percentage of 65% (see Table 4).

**Table 4:** An example of scratch card information provided to participants in experiment 3

Card type	Game	Prize amount	Total prizes	Unclaimed prizes
High unclaimed prizes	100X Multiplier (green)	$1,000,000	2	2
	100X Multiplier (green)	$25,000	10	7
	100X Multiplier (green)	$1000	100	78
	Total number of tickets remaining: 693,231			
Medium unclaimed prizes	100X Multiplier (blue)	$1,000,000	2	1
	100X Multiplier (blue)	$25,000	10	5
	100X Multiplier (blue)	$1000	100	54
	Total number of tickets remaining: 336,857			
Low unclaimed prizes	100X Multiplier (red)	$1,000,000	2	0
	100X Multiplier (red)	$25,000	10	3
	100X Multiplier (red)	$1000	100	24
	Total number of tickets remaining: 26,400			

## Results

### Likelihood of Winning

**Fig. 3:**
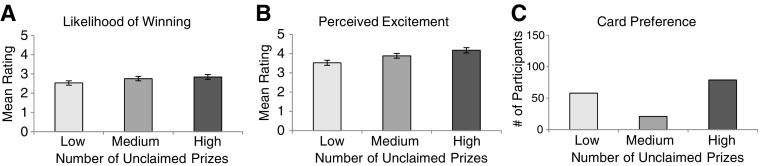
Results from Experiment 3. **a** Mean likelihood of winning values for low, medium, and high unclaimed prize levels. **b** Mean perceived excitement values for low, medium, and high unclaimed prize levels. **c** Mean number of cards chosen for low, medium, and high unclaimed prize levels. All error bars are ± 1 SE

Participants’ ratings of how likely a win was on the various 100X Multiplier cards were averaged and compared with a repeated measures analysis of variance (ANOVA). The overall ANOVA revealed a main effect of unclaimed prize information, *F*(1.45, 281.92) = 4.68, *p* = .019, $\eta _{p}^{2}$ = .024 (see Fig. 3a).^1^ Follow-up paired samples * t*-tests revealed a significant difference between participant’s likelihood ratings for games featuring a high number of unclaimed prizes (*M* = 2.84, SD = 1.79) and a low number of unclaimed prizes (*M* = 2.53, SD = 1.59), *t*(194) = 2.35, *p* = .020, as well as a significant difference between likelihood ratings for games featuring a medium number of unclaimed prizes (*M* = 2.75, SD = 1.58) and a low number of unclaimed prizes, *t*(194) = 2.30, *p* = .023. No significant difference was found when comparing likelihood ratings given to cards featuring high and medium amounts of unclaimed prizes (*p *> .05). We once again sought to compare participants’ likelihood ratings given to each version of 100X Multiplier (featuring either high, medium, or low unclaimed prize information) with baseline likelihood ratings (made prior to the presentation of unclaimed prize information). Replicating the results of Experiment 2, likelihood ratings given to scratch cards featuring low amounts of unclaimed prizes were revealed to be significantly lower than participants’ baseline ratings (*M *= 2.90, SD = 1.63), *t*(197) = − 3.52, *p* = .001. All other comparisons with participants’ baseline likelihood ratings failed to reach significance (*p* > .05).

### Perceived Excitement

Participants’ ratings of perceived excitement for playing the given scratch card games were averaged and compared with a repeated measures ANOVA. The overall ANOVA revealed a main effect of unclaimed prize information, *F*(1.54, 300.70) = 17.17, *p* < .001, $\eta _{p}^{2}$ = .081 (see Fig. 3b). Follow-up paired samples *t*-tests revealed significant differences between excitement ratings when there was a high number of unclaimed prizes (*M* = 4.17, SD = 1.91) compared to when their was a low (*M* = 3.52, SD = 1.88), *t*(195) = 4.74, *p* < .001, or medium amount of unclaimed prizes (*M* = 3.88, SD = 1.73), *t*(195) = 3.42, *p* = .001. Additionally, a significant difference was revealed between excitement ratings given to cards featuring medium amounts of unclaimed prizes and cards featuring low amounts of unclaimed prizes, *t*(195) = 3.42, *p* = .001.

### Card Preference

As in Experiments 1 and 2, participant’s card preferences were assessed by determining which card each participant preferred (i.e., the card that they hypothetically purchased the most). Forty-two participants in Experiment 3 failed to establish a clear preference in favour of one card (i.e., split their preference between two cards equally) and therefore were not included in our analyses. Of the remaining 158 who did establish a clear card preference, 79 preferred to hypothetically purchase cards with a high number of unclaimed prizes. Twenty-one participants showed a preference for scratch cards with a medium number of unclaimed prizes, and 58 participants showed a preference for scratch cards with a low number of unclaimed prizes (see Fig. 3c). To compare the number of cards purchased between each of the unclaimed prize categories, we conducted Chi square goodness-of-fit tests, examining the number of participants who established a card preference (calculated for each card type), against a theoretical expected number of preferred cards assuming no participant preferences (i.e., equal preference between all card types involved in the analysis).

First, we conducted a test to determine if the observed preferences among all three card types differed from the preferences that would be expected if participants were not influenced by unclaimed prize information in our hypothetical purchasing task. This test revealed an influence of unclaimed prize information as observed preferences significantly differed from the preferences one would expect given no influence of unclaimed prizes, *X*^2^(2) = 32.75, *p* < .001. Next, we conducted three further Chi square goodness-of-fit tests comparing participants’ preferences for high versus medium, high versus low, and medium versus low unclaimed prize cards, compared to equal preferences between these pairs. These analyses revealed that participants preferred cards with a high amount of unclaimed prizes compared to cards featuring a medium amount of unclaimed prizes, *X*^*2*^(1) = 16.82, *p* < .001, however, participants’ choices did not demonstrate a preference for cards featuring high over low unclaimed prize amounts, *X*^2^(1) = 1.61, *p* > .05. Furthermore, participants preferred cards with low numbers of unclaimed prizes compared to those with medium numbers of unclaimed prizes, *X*^*2*^(1) = 8.66, *p* < .01.

### Willingness to Pay

The median willingness to pay value reported was $5.00, and responses ranged from $0.00 to $12,000. Importantly, only 28 participants (14.5%) indicated that they would not pay for unclaimed prize information (i.e., input $0 as their response).

### Calculating Payback Percentage

One hundred and forty-two participants (71.4%) self-reported that, given the necessary information, they would be able to correctly calculate a scratch card’s payback percentage. However, when asked to demonstrate this ability, only 31 participants (15.5%) were able to report the correct payback percentage of a presented scratch card game.

## Discussion

Although the information provided to participants was constructed such that scratch cards featuring fewer unclaimed prizes had an overall higher payback percentage, participants still preferred scratch cards with more unclaimed prizes despite their lesser value. This preference was demonstrated by participants’ assessment of their likelihood of winning, perceived excitement, and card preferences in a hypothetical card purchasing scenario. Furthermore, the magnitude of bias due to unclaimed prize information does not appear to be reduced in Experiment 3 compared to Experiment 2 (see Table 5). This suggests that participants’ judgments were not sensitive to our payback percentage manipulation.

**Table 5:** A comparison of effect sizes between experiments

	Experiment 1	Experiment 2	Experiment 3
Likelihood of winning	.259	.018	.024
Perceived excitement	.210	.047	.081

Effect size as represented by $\eta _{p}^{2}$

## General Discussion

Across three experiments, we demonstrated that people are biased by the presence of unclaimed prize information. In Experiment 1, participants felt more likely to win, were more excited to play, and chose to hypothetically purchase scratch cards with higher levels of unclaimed prizes. In Experiment 2, with the addition of ticket remaining information, unclaimed prize information continued to bias participants’ judgments despite the fact that payback percentage could be calculated and utilized for each scratch card. Finally, in Experiment 3, this bias persisted when the payback percentage of each card was put into direct conflict with unclaimed prize information, such that participants’ best card choice was the counterintuitive *low* unclaimed prize card. Along with being influenced by unclaimed prize information, the majority of participants across all three experiments stated that they would be willing to pay five or more dollars for this information, further demonstrating that many believed that this information was useful for deciding between scratch card games.

In the present studies, participants displayed preferences that parallel the preferences observed in ratio bias tasks, such that their judgments were influenced by the intuitively appealing, yet uninformative unclaimed prize information. Also consistent with the literature on ratio bias, participants’ preferences for scratch cards with a greater number of unclaimed prizes remained when additional information was provided so that an informative ratio could be created. Therefore, one explanation for the biasing effects of unclaimed prize information is that the salience and intuitive appeal of this information results in this information being favoured when in conflict with other pieces of informative, yet less intuitive, information (e.g., the ratio of unclaimed prizes to total number of tickets remaining).

Research examining conflict detection in ratio bias tasks suggests that although participants often choose the intuitively appealing alternative, they readily detect the conflict between presented alternatives (Bonner and Newell 2010; Denes-Raj and Epstein 1994). Therefore, ratio bias does not appear to occur as a result of participants’ failure to consider ratio information. Consistent with this claim, participant self-report data suggests that even when aware that their intuitive choice is suboptimal, this alternative still appears attractive and is frequently chosen by participants (Denes-Raj and Epstein 1994). These previous findings may shed light on our current results, such that our attenuated but persisting unclaimed prize information bias may be due to the fact that although participants appear to attend to ticket remaining information in Experiments 2 and 3, they preferred to make their choices on the basis of the more intuitively appealing unclaimed prize information.

Another potential explanation for the persistent biasing effects of unclaimed prize information in Experiments 2 and 3 may be participants’ inability to properly incorporate both unclaimed prize and ticket remaining information. In support of this explanation, the majority of participants demonstrated their inability to properly calculate payback percentage (i.e., the value of all unclaimed prizes divided by the value of all tickets remaining). Thus, unclaimed prize information bias may simply be a result of the majority of participants lacking the tools necessary to use the presented information in a useful way.

Given the persistent effects of this information on participants’ judgments, one may ask why unclaimed prize information is presented to scratch card players in the first place. Past annual reports from the Ontario Lottery and Gaming Corporation suggest that this information may be provided to players out of legal obligation (Ontario Lottery and Gaming Corporation 2009), to inform players that specific advertised grand prizes may not actually be available to be won. While useful in this regard, this series of studies clearly demonstrates an unintended consequence of presenting such information to players, namely its strong impact on participants’ judgments within a hypothetical gambling scenario. This highlights the importance of investigating the influence of information that is presented in real-world gambling contexts, especially information that may appear intuitively useful, but is otherwise uninformative.

## Limitations and Future Directions

One limitation of our study design is that the low unclaimed prize scratch card did not contain any unclaimed top prizes. However, in Experiments 2 and 3, this discrepancy was remedied by an equally, and at times more extreme correction in the form of ticket remaining information. Additionally, we frequently observed the biasing effects of unclaimed prize information between the medium and high levels of unclaimed prize information. Future experiments could address this limitation by utilizing different combinations of unclaimed prizes. Furthermore, participants only engaged in hypothetical gambling scenarios during the experiment. Future studies should examine the effect of unclaimed prize information in a real-world gambling context, to generalize the applicability of these results. Lastly, it is still an open question whether or not the use of this information makes individuals gamble more frequently, or encourages more problematic gambling behaviour; future studies could attempt to elucidate these effects.

Moreover, it may be beneficial to investigate different ways of reducing the influence of unclaimed prize information, possibly with messaging or disclaimers. Another possible avenue for reducing the effects of this information is teaching people how to properly use it, whether this be calculating payback percentage when possible, or ignoring the information when it is presented independently (as is common in real-world gambling contexts).

## Conclusion

Unclaimed prize information is a readily available source of information provided to players by lottery operators. Our series of three experiments exemplifies the potential unintended consequences of unclaimed prize information: participants feel more likely to win, are more excited to play, and more frequently purchase hypothetical scratch cards with greater amounts of unclaimed prizes. This bias persists even when participants are provided with additional information allowing them to more accurately assess the value of a scratch card (i.e., by calculating payback percentage). These results suggest that participants may be unduly influenced by the seemingly highly intuitive and appealing unclaimed prize information, even in the presence of more informative information, or may lack the tools necessary to utilize these various pieces of information in a useful way.

## Footnotes

1 With non-naïve participants removed, the overall repeated measures ANOVA revealed no main effect of unclaimed prize information on likelihood of winning judgments, *F*(1.48, 226.23) = 1.53, *p* = .221, $\eta _{p}^{2}$ = .010. However, the likelihood of winning judgments for both non-naïve and naïve participants exhibited the same relationship across the levels of unclaimed prizes (e.g. highest likelihood of winning judgments for the highest unclaimed prize level; lowest likelihood of winning judgments for the lowest unclaimed prize level).
